# Characterization of ten novel Ty1/*copia*-like retrotransposon families of the grapevine genome

**DOI:** 10.1186/1471-2164-9-469

**Published:** 2008-10-09

**Authors:** Cédric Moisy, Keith E Garrison, Carole P Meredith, Frédérique Pelsy

**Affiliations:** 1 INRA, UMR1131, F-68000, Colmar, France; 2 Université de Strasbourg, UMR1131, F-67000, Strasbourg, France; 3 Department of Viticulture and Enology, University of California, Davis, California, 95616, USA; 4 Department of Biology, St. Mary's College of California, Moraga, California, 94556, USA

## Abstract

**Background:**

Retrotransposons make a significant contribution to the size, organization and genetic diversity of their host genomes. To characterize retrotransposon families in the grapevine genome (the fourth crop plant genome sequenced) we have combined two approaches: a PCR-based method for the isolation of RnaseH-LTR sequences with a computer-based sequence similarity search in the whole-genome sequence of PN40024.

**Results:**

Supported by a phylogenic analysis, ten novel Ty1/*copia *families were distinguished in this study. To select a canonical reference element sequence from amongst the various insertions in the genome belonging to each retroelement family, the following screening criteria were adopted to identify the element sequence with: (1) perfect 5 bp-duplication of target sites, (2) the highest level of identity between 5' and 3'-LTR within a single insertion sequence, and (3) longest, un-interrupted coding capacity within the *gag-pol *ORF. One to eight copies encoding a single putatively functional *gag-pol *polyprotein were found for three families, indicating that these families could be still autonomous and active. For the others, no autonomous copies were identified. However, a subset of copies within the presumably non-autonomous families had perfect identity between their 5' and 3' LTRs, indicating a recent insertion event. A phylogenic study based on the sequence alignment of the region located between reverse transcriptase domains I and VII distinguished these 10 families from other plant retrotransposons. Including the previously characterized *Ty1*/*copia*-like grapevine retrotransposons *Tvv1 *and *Vine 1 *and the Ty3/*gypsy*-like *Gret1 *in this assessment, a total of 1709 copies were identified for the 13 retrotransposon families, representing 1.24% of the sequenced genome. The copy number per family ranged from 91–212 copies. We performed insertion site profiling for 8 out of the 13 retrotransposon families and confirmed multiple insertions of these elements across the *Vitis *genus. Insertional polymorphism analysis and dating of full-length copies based on their LTR divergence demonstrated that each family has a particular amplification history, with 71% of the identified copies being inserted within the last 2 million years.

**Conclusion:**

The strategy we used efficiently delivered new Ty1/*copia*-like retrotransposon sequences, increasing the total number of characterized grapevine retrotrotransposons from 3 to 13. We provide insights into the representation and dynamics of the 13 families in the genome. Our data demonstrated that each family has a particular amplification pattern, with 7 families having copies recently inserted within the last 0.2 million year. Among those 7 families with recent insertions, three retain the capacity for activity in the grape genome today.

## Background

Sequencing of whole genomes reveals the predominant amount of transposable element DNA they contain, as well as how transposable elements have shaped the genome. Four plant genomes are now fully sequenced: Arabidopsis [1], rice [2], black cottonwood [3] and more recently two grapevine cultivars [4,5]. Annotation revealed that the amount of repetitive DNA depended on the species, with repetitive DNA composing ~10% of the Arabidopsis genome (125 Mbp), ~35% of rice genome (389 Mbp), and ~41.4% of the grapevine genome (487 Mbp). Analysis of repeated DNA sequences carried out in these genomes, as well as in larger cereal genomes not yet fully sequenced, has shown that insertion of long terminal repeat (LTR) retrotransposons of the two main superfamilies, Ty3/*gypsy*-like and Ty1/*copia*-like are the major components of intergenic regions.

Retrotransposons are mobile elements that are closely related to retroviruses in their structure and life cycle [6]. Active retrotransposons begin the process of transposition when RNA is transcribed from the original retrotransposon insertion in the genome. The intermediate RNA transcript is reverse-transcribed into DNA by a retrotransposon-encoded reverse transcriptase enzyme, and the DNA is inserted by additional retrotransposon-encoded enzymes into the host genome at a new location, thus increasing the copy number of the retrotransposon family [7]. The generation and reverse-transcription of the RNA intermediate is what distinguishes retrotransposons from DNA transposons such as the *Ac*/*Ds *elements in maize, which are excised by transposon-encoded enzymes from their original insertion site and moved directly to a new insertion site without an RNA intermediate [8,9]. The copy-and-paste cycle of retrotransposition in the host genome requires the synthesis of specific proteins encoded by two major retrotransposon genes, *gag *and *pol*. The Ty1/*copia *[10,11] and the Ty3/*gypsy *[12] superfamilies differ from each other in the order of domains within the *pol *gene, as well as in their degree of sequence conservation. Specifically in Ty1/*copia *elements, the *pol *gene encodes the protease, integrase, reverse transcriptase (RT), and RnaseH domains in that order from the 5' end of the gene. The enzymatic functions encoded in each of these domains are required to synthesize an extrachromosomal DNA daughter copy by reverse-transcription from the intermediate RNA prior to its reinsertion into the genome. As the process of reverse transcription is highly error-prone due to the lack of proofreading, the daughter copies can differ from the master copy [13]. Moreover, individual copies can also diverge independently by mutation following their insertion into the genome. Therefore, one hallmark of retroelements is the genetic heterogeneity of individual copies belonging to the same family [14].

Despite the heterogeneity within retrotransposon families, effective grouping of elements into phylogenetically-supported clusters of related insertions followed by assessment of the level and timing of divergence within those clusters is necessary for informative analysis. The RT domain has the slowest relative rate of change among all retroelement proteins, while the *gag *domain has a rate of change more than twice as high [15]. Because of the extent of sequence conservation within the reverse transcriptase protein, the use of sequence comparisons of reverse transcriptase domains I and VII has been proposed to group retrotransposons into a family when the amino acid identity with other members is ≥90% and into a superfamily when their identity is ≥75% [16]. While these are effective criteria for grouping individual insertions into meaningful clusters, better methods exist for determining the age of an individual insertion in the genome. Because of the mechanism of transposition, the two LTRs at each end of a retrotransposon are theoretically identical at the time of their insertion [17-19]. After insertion, the 5' and 3' LTRs begin to evolve independently and diverge from each other in sequence. Thus, the sequence divergence between the 5' and 3' LTRs of an individual element can be used to estimate the time since insertion at a specific locus [20].

Various strategies have been employed to identify retrotransposon sequences in genomes. Prior to the determination of the complete genomic sequence of the organism, full-length or parts of Ty1/*copia*-like retrotransposon were identified by virtue of their presence within coding sequence being characterized, such as *Tnt1 *in the tobacco nitrate reductase gene [21] or *Vine-1 *in a grape *Adh*r gene [22]. Others insertions have been identified through their contribution to an important phenotypic character such as the color of grape skins associated with the presence of the *Gret1 *retrotransposon in the promoter region of *VvMybA1 *[23]. Following the completion of genomic sequencing of *Arabidopsis*, retroelement sequences have also emerged from computer-based sequence similarity searches in genomic databases [24]. Few techniques exist to identify explicitly retrotransposon sequences in genomes prior to the characterization of the complete genome sequence for an organism. To address this need, Pearce et al. developed a PCR-based method to identify experimentally Ty1/*copia*-like retrotransposon LTR sequences of any higher plant, and verified its utility by isolating novel LTR sequences from pea, broad bean and Norway spruce [25]. Following this procedure, Riccioni et al. have isolated *Tmt1*, the first LTR-retrotransposon from ectomycorrhizal fungi of the genus *Tuber *that shows relatedness to Ty3/*gypsy *retrotransposons [26].

Because retrotransposons make a significant contribution to the size, organization and genetic diversity of genomes, characterization of grapevine retrotransposons is important. Three retrotransposons were described prior to the grape whole-genome sequencing: a unique copy of *Vine-1*, a Ty1/*copia*-like element [22] and of *Gret1*, a Ty3/*gypsy*-like element [23] and the *Tvv*1 family [27,28]. The grape genome sequence has recently been obtained from two sources: a highly homozygous grape line PN40024 [4] and the Pinot noir cultivar [5]. These genomic resources make possible the characterization *in silico *of grape retrotransposons. Annotation of grapevine transposable elements in the sequence derived from both sources revealed a large prevalence of retrotransposons over transposons. In the PN40024 sequence, the most abundant transposable elements corresponded to Ty1/*copia*-like retrotransposons, with an uneven distribution over the grape chromosomes, and mainly restricted to low gene density regions and introns [4]. In contrast, within the Pinot noir genomic sequence the most prevalent retrotransposons corresponded to *gypsy*/*athila*-like elements [5]. In this study, we extend the utility of the Pearce technique into organisms with characterized, complete genomes. We first discovered novel Ty1/*copia*-like families in the grapevine genome through analysis of RnaseH-LTR sequences isolated by the Pearce technique from genomic DNA derived from Pinot noir plant material. We then used the isolated sequences as a highly diverse query set to perform a computer-based sequence similarity search against the completed grape genome sequence dataset. Supported by phylogenic analysis, we distinguished novel families and identified a canonic representative element for each family. Their phylogenic relationship with other reference plant retrotransposons was studied. Based on LTR divergence, insertion dating of full-length copies belonging to 13 grapevine retrotransposon families was estimated in order to study the dynamics of these families. Finally, the sequence-specific amplification polymorphism (S-SAP) profiles [29] of 8 retrotransposon families were evaluated in 10 *Vitis *accessions.

## Results

### Characterization of ten new Ty1/*copia*-like retrotransposon families of the grapevine genome

Our application of the technique of Pearce et al. [25] to the grapevine genome resulted in the isolation of 27 putative retrotransposon sequences. Among these putative retrotransposon sequences, 24 contained an identifiable portion of the 3'-end of Ty1/*copia-*like retrotransposons including a putative RnaseH domain, the polypurine tract (PPT), and varying amounts of the 3'LTR sequence. Eighteen of them were unique and six almost identical (subsequently characterized as *Edel *sequences). None of the fragments we identified were similar enough to one of the three grapevine retrotransposon families previously characterized to be considered additional insertions within those previously characterized families.

**Figure 1:**
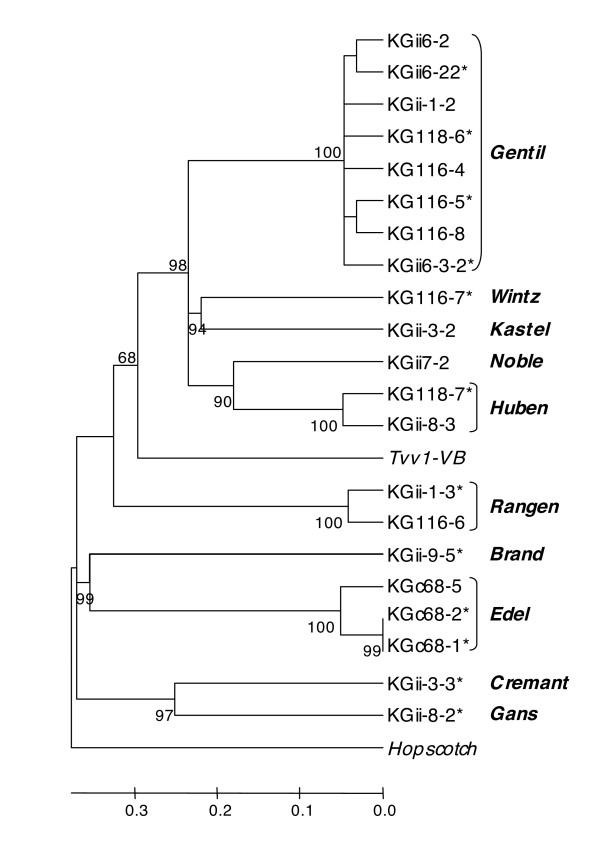
**Classification of the 21 novel full-length retrotransposon sequences into 10 families**. The Neighbor-Joining tree is based on the multiple sequence alignment of amino acid sequences between reverse transcriptase domains I and VII using the Poisson distance model. * Insertions displaying perfect target duplication sites. Ty1/*copia *retrotransposons *Tvv1 *from grapevine and *Hopscotch *from maize (acc. n° U12626) were added as references. Bootstrap values greater than 60% are shown on the tree.

A data mining procedure from the grape genomic sequence using the BLAST algorithm with the 18 unique and 3 of the nearly identical sequences as queries yielded 21 different full-length retrotransposons harboring an internal domain flanked by LTRs, twelve of them displaying perfect target duplication sites. Thus, the 3'-end sequences of the retrotranpsosons identified by the Pearce technique could be used to 'tag' a diverse set of full- or nearly full-length retrotransposons within the completed grape genome. A phylogenic analysis was conducted based on the alignment of the amino acid sequence located between reverse transcriptase domains I and VII deduced from the upstream nucleic sequence of the 21 elements. *Tvv1-VB *[28], one of the two Ty1/*copia-*like retrotransposons previously characterized in the grapevine genome were added to improve the comprehensiveness of this analysis. The other, *Vine 1*, could not be included in this analysis, as it lacks the RT-domain sequence that forms the basis for this comparison. The Neighbor-Joining tree clustered the 21 elements into 10 distinct families, which were also distinct from *Tvv1-VB *(Figure 1). The largest family, *Gentil*, included eight elements, the *Edel *family three elements and two families, *Huben *and *Rangen*, each included two elements. Finally, six families, *Brand, Cremant, Gans, Kastel, Noble *and *Wintz *were represented by a single element. The domain order inside the *pol *gene of the amino acid sequence suggested that all the novel families identified by the Pearce technique were Ty1/*copia*-like elements. The clustering data were confirmed by evaluating the identity level of the partial RT domains as suggested by Bowen and McDonald [16]. This level was ≥79% within elements classified in the same family, and ranged from 35–67% for elements classified into distinct Ty1/*copia *families. The identity level of the partial domains ranged from 12–21% for comparisons between *Gret1*, a Ty3/*gypsy *element, and the various Ty1/*copia *families.

### Representation of the 13 families in the grapevine genome

The BLAT program was used to extract the paralogous copies of the ten families from the grapevine genome along with *Tvv1-VB*, *Vine-1 *and *Gret1*. A total of 1709 sequence matches were identified, with the copy number per family ranging from 91 to 212. The families with the highest copy numbers were *Gentil *(212), *Edel *(187), *Noble *(185), and *Gret1 *(173) while *Huben *and *Kastel *(91) had the lowest copy number (Additional file 1). For all families except *Gret1*, copies longer than the reference size were found. In particular, one *Edel *element contains a long insertion that extends its size to over 382% of the reference copy. Generally, all families had a median length below that of the reference query, indicating that most of the copies were truncated, but with a high sequence identity to the reference sequence (median range 0.80–0.97). Using *Vine-1 *as the query, most of the additional insertions also correspond to truncated copies. One larger copy was 107.6% of the reference *Vine-1 *copy length, but the additional sequence in this insertion does not code for the RT-RnaseH domain that is lacking in the reference *Vine-1 *element [22].

Considering that all retrieved copies likely were equal in length to their corresponding reference sequences at the time of their insertion, they would represent a total theoretical DNA quantity of 8.9 Mb added to the genome by their specific amplification. The *Gret1 *family, because of a reference copy size of 10,422 bp, and *Noble*, due to a combination of large reference copy size and high genomic copy number among the Ty1/*copia *families in the grape genome, would likely have contributed the greatest to the retroelement DNA content. Summing all actual retroelement insertion lengths, a residual amount of 4.8 Mb of retrotransposon DNA (representing 1.24% of the PN40024 genome) remains in the genome, suggesting that 47% of retrotransposon inserted sequence has been removed between the time of insertion and the present day. Copies classified into the 13 families are scattered around all chromosomes as currently assembled with an overall density of 4.43 copies per Mb (Table 1). However, this distribution is not uniform. With 54 copies, chromosome 8 (21.56 Mb) has the lowest retrotransposon insertion density (2.50 insertion per Mb) while chromosome 16 (8.159 Mb) has a density almost a third higher (7.23 insertion per Mb). Most retrotransposon families seem to be rather uniformly distributed on all chromosomes except *Gans*, which is absent from 6 chromosomes but present in 17 copies on chromosome 5.

**Table 1:** Copy density on the 19 current chromosomes

**Location PN40024**	**Total copy number**	**Chromosome size (Mb)**	**Density (copy/Mb)**
chr 1	73	15.63	4.67
chr 2	57	17.6	3.24
chr 3	33	10.19	3.24
chr 4	60	19.29	3.11
chr 5	86	23.43	3.67
chr 6	69	24.15	2.86
chr 7	50	15.23	3.28
chr 8	54	21.56	2.50
chr 9	68	16.53	4.11
chr 10	63	9.647	6.53
chr 11	52	13.94	3.73
chr 12	91	18.54	4.91
chr 13	61	15.19	4.02
chr 14	95	19.48	4.88
chr 15	51	7.694	6.63
chr 16	59	8.159	7.23
chr 17	63	13.06	4.82
chr 18	122	19.69	6.20
chr 19	64	14.07	4.55
Unknown	438		

### Identification of the canonical copies

For each family, a canonical copy was identified among elements whose length was at least 80% of the full-length insertions identified by BLAT with an identity level higher than 75%. The following additional criteria helped to identify the canonical copy: (1) perfect 5 bp-duplication of target sites, (2) the highest level of identity between 5' and 3' LTR within a single insertion sequence, and (3) longest, un-interrupted coding capacity within the *gag-pol *ORF. These criteria are likely to identify the most recently inserted and potentially active member of the family. The criteria differ significantly from the consensus approach that identifies ancestral, presumably active sequences within retroelement families [30]. The 10 canonical elements selected varied in size from 4,086 to 5,519 bp, with LTRs ranging from 147 to 502 bp (Additional file 2). For eight of the families, LTRs begin and end with the dinucleotide inverted repeat TG...CA as observed for many LTR-retrotransposons and retroviruses [31]. More precisely, seven start with the four-nucleotide stretch TGTT, and among them two, *Brand *and *Rangen*, end with the corresponding inverted stretch AACA. *Huben *and *Wintz *LTRs begin with the four-nucleotide stretch TGTG and end with TTCA and GTCA, respectively. Finally, *Cremant *LTRs begin with the four-nucleotide stretch TAAC and end with GTCA.

One *Cremant*, 3 *Edel *and 8 *Noble *elements showed a single putative ORF uninterrupted by a stop-codon before the PPT, corresponding to polyproteins that were 1,346, 1,298 and 1,486 amino acids long respectively, indicating that they could be autonomous. By comparison with *Ty1/copia-*like plant retrotransposons previously characterized, the general organization of the putative ORFs of *Cremant, Edel *and *Noble *elements is very similar both in structure and in length, compared to the 1,296, 1,382, 1,328 and 1,447 amino acid-long ORF of the barley *BARE-1 *(acc. n° Z17327), grapevine *Tvv1*-*VB *(acc. n° EU304807), tobacco *Tnt1 *(acc. n° X13777), and *Arabidopsis thaliana AtRE1 *(acc. n° AB021265) retrotransposons, respectively. The putative ORFs of the seven remaining elements were prematurely interrupted by a stop codon or a frameshift in the coding regions. Although *Gans*, *Gentil, Huben *and *Wintz *canonic copies lack a putative full-length polyprotein, the homology between LTRs (100%, 99.7%, 100% and 99.3%, respectively) indicates that they moved recently into their current insertion site (Additional file 2). The 8 *Noble *copies that are putatively active displayed an ORF 4,458 bp-long but differed by their untranslated leader (UTL) region located between the 5'LTR and the ORF, which ranged in size from 578 to 632 bp-long. Moreover, considering the 40 full-length *Noble *copies identified by perfect host 5 bp-target sites, their UTL region varied from 269 bp to 695 bp (average 610 bp), and all started with the sequence TGGTATCAGAGCC which corresponds to the PBS. A detailed analysis of the UTL sequence within 12 *Noble *elements indicated that variations in size of this region have essentially resulted from large single deletions/insertions.

**Figure 2:**
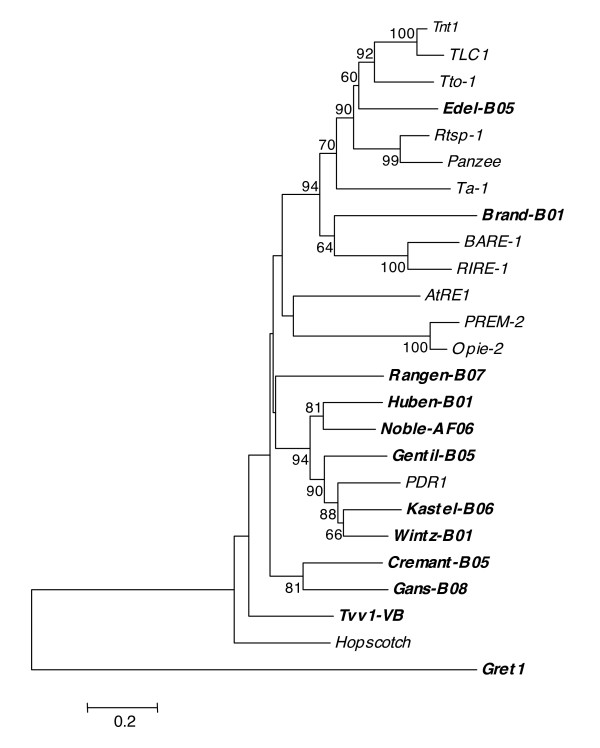
**Phylogenetic analysis of the 10 novel families**. The Neighbor-Joining tree is based on the multiple sequence alignment of amino acid sequences between reverse transcriptase domains I and VII using the Poisson distance model. For novel families, amino acid sequences were deduced from canonic copies and for other plant retrotransposons from published nucleotide sequences:*A. thaliana AtRE1*; *A. thaliana Ta-1 *(acc. n° X13291); *Cajanus cajan Panzee *(acc. n° AJ000893); *Hordeum vulgare BARE-1*; *Ipomoea batatas Rtsp-1 *(acc. n° AB162659); *Nicotiana tabacum Tnt1*; *N. tabacum Tto1 *(acc. n° D83003); *Oryza sativa RIRE-1 *(acc. n° D85597); *Pisum sativum PDR1 *(acc. n° X66399); *Solanum chilense TLC1 *(acc. n° AF279585); *V. vinifera Tvv1*-*VB*; *V. vinifera Gret1 *(acc. n° AB111100);*Zea mays PREM-2 *(acc. n° U41000); *Z. mays Opie-2 *(acc. n° AF090446). Grapevine retrotransposon families are given in bold. Bootstrap values greater than 60% are shown on the tree.

To elucidate the phylogenic relationships among the ten novel families, *Tvv1, Gret1*, and 13 other plant *Ty1/copia*-like retrotransposons, the amino acid sequences between motifs I to VII of the RT were aligned [32]. In some cases, grape retrotransposon families clustered more closely with previously identified retrotransposon families in other species than with other retrotransposon families in grape (Figure 2).

### Genomic paleontology

**Figure 3:**
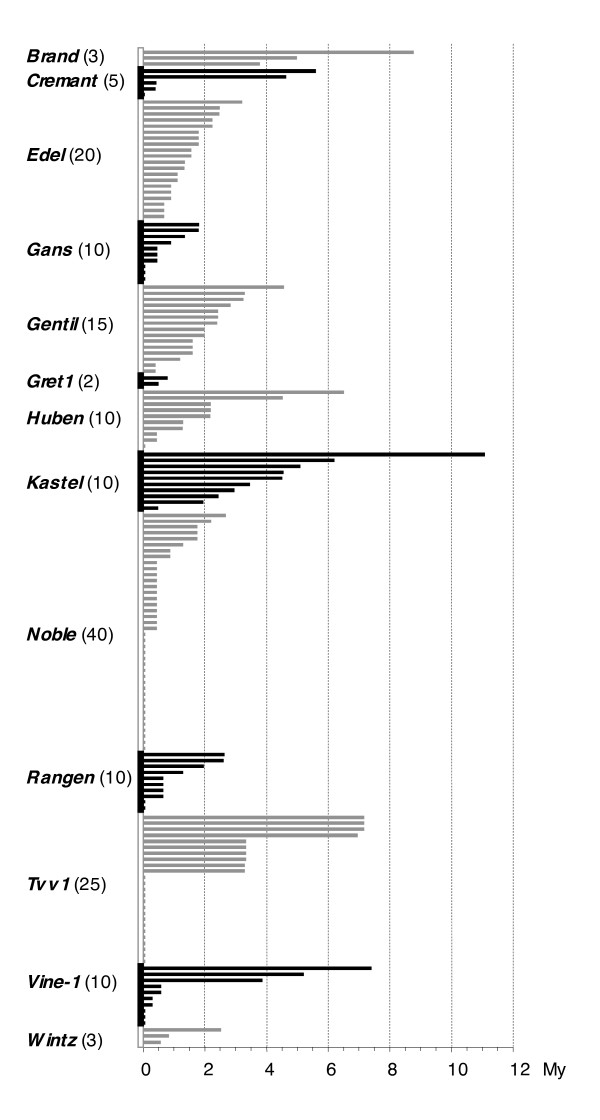
**Distribution of the insertion dates for full-length copies belonging to the 13 grapevine LTR retrotransposon families**. LTR divergence was used to estimate date of insertion of full-length copies that were also flanked by perfect direct repeats. The copy number of full-length elements for each family is given in parenthesis.

The insertion dates for 163 retrotransposon copies considered to be full-length and belonging to the 13 families of this study (the 10 newly identified along with *Gret1*, *Vine-1*, and *Tvv1*) were estimated by aligning the 5' and 3' LTR sequence according to Vitte et al. [33]. This analysis (Figure 3) revealed that copy age ranged from ≤0.2–11 million years (My). One hundred and fifteen copies (70%) were inserted within the last 2 My. Forty-five copies belonging to 7 families (28% of the total number of full-length copies) had identical 5' and 3'-LTRs, indicating recent movement at the limit of detection (0% divergence theoretically corresponding to an insertion within the last 0.2 My) for this analysis. Two types of insertion age patterns within retroelement families were obtained, reflecting distinct evolutionary histories and activities of particular families. Some families had a continuous distribution of insertion dates such as *Edel *(1–5 Mya) or *Gentil *(1–7 Mya). In contrast, other families show discrete groups of copies inserted at the same date such as *Tvv1 *(recent, 3 and 7 Mya) or *Noble *(recent and 0.5 Mya) possibly reflecting different bursts of amplification.

### Insertion pattern of 8 retrotransposon families within the genus *Vitis*

**Figure 4:**
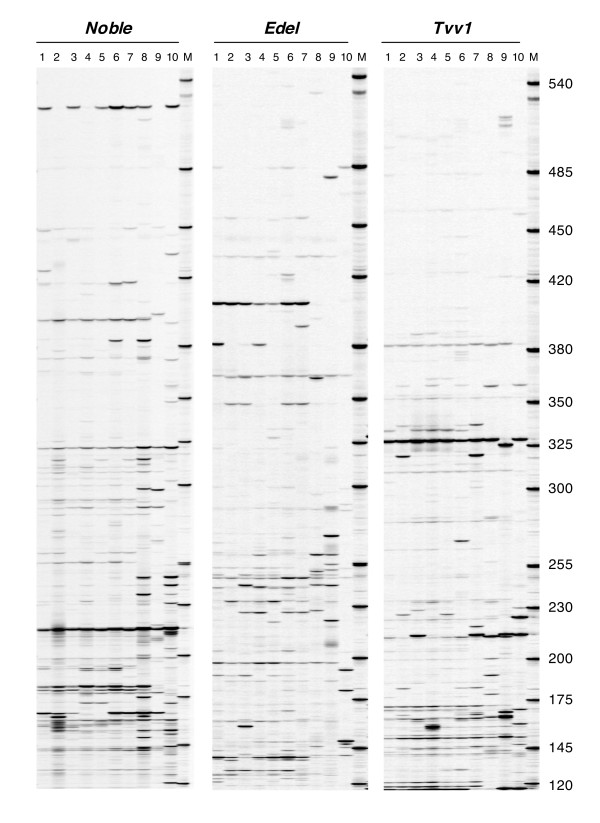
**Comparison of S-SAP insertion patterns of 4 retrotransposons on 10 *Vitis *accessions**. Accessions are numbered as followed: 1: PN40024, 2: pinot noir, 3: cabernet sauvignon, 4: grenache, 5: muscat d'Alexandrie, 6: syrah, 7: *Vitis silvestris*, 8: *Vitis amurensis*, 9: *Vitis riparia*, 10: *Muscadinia rotundifolia*.

To visualize the distribution of copies belonging to 8 of the retrotransposon families across the *Vitis *genus, a set of 10 *Vitis *accessions including a subset of 7 accessions of the *V. vinifera *species was chosen. For each retrotransposon family, S-SAP insertion patterns differed both by the number of scored bands and by the polymorphism level for all accessions (Figure 4). In the *Vitis *set, the 8 patterns yielded a total of 31 to 70 scored bands ranging in size from 125–573 bp, depending on the family (Table 2). This number decreased to 19–50 scored bands within the *V. vinifera *subset. In both sets, the lowest number of scored S-SAP bands was obtained for *Edel *and the highest for *Huben*. The percentage of polymorphic bands ranged from 85.7–100% within the *Vitis *set and from 54.5–94.7% within *V. vinifera *subset. The highest number of bands shared by accessions in both sets corresponded to *Gans *insertions while only one *Edel *insertion was common to the 7 *V. vinifera *accessions.

**Table 2:** Genetic variability values of the S-SAP fingerprinting

	**10 *Vitis *accessions**			**7 *V. vinifera *accessions**		
	
**Scored band number**	**Total**	**Polymorphic**	**Monomorphic**	**Total**	**Polymorphic**	**Monomorphic**
***Edel***	31	31 (100%)	0	19	18 (94.7%)	1 (5.3%)
***Gans***	42	36 (85.7%)	6 (14.3%)	40	26 (65.0%)	14 (35.0%)
***Gentil***	57	54 (94.7%)	3 (5.3%)	45	39 (86.7%)	6 (13.3%)
***Gret1***	44	43 (97.7%)	1 (2.3%)	33	30 (90.9%)	3 (9.1%)
***Huben***	70	67 (95.7%)	3 (4.3%)	50	41 (82.0%)	9 (18.0%)
***Noble***	33	30 (90.9%)	3 (9.1%)	22	12 (54.5%)	10 (45.5%)
***Tvv1***	35	35 (100%)	0	21	17 (81.0%)	4 (19.0%)
***Vine-1***	59	57 (96.8%)	2 (3.4%)	45	38 (84.4%)	7 (15.6%)

S-SAP data were obtained using forward primers designed from 8 retrotransposons in combination with the same Eco-AG adapter primer.

## Discussion

### Identification of the novel retrotransposon families

Combining an RnaseH-LTR analysis of sequences isolated using the technique described by Pearce et al. [25] with a computer-based sequence similarity search in the whole-genome grapevine sequence, we have identified ten novel Ty1/*copia*-like retrotransposon families. None of the sequences identified in this study belong to a grapevine retrotransposon family previously described. This result demonstrates effective complementation of bench laboratory and in silico techniques, enhancing the benefits of either used alone. If standard laboratory cloning techniques (e.g. genome 'walking') were used to obtain complete retrotransposon sequence from the 3'-end sequences isolated by the Pearce technique, considerable time and expense would have been devoted to the subsequent procedures and necessary reagents. Full-length elements were effectively cloned *in silico *using the sequences identified by the Pearce technique as a query for genomic searches. However, if a purely *in silico *approach had been employed to isolate new retrotransposon sequence from grape using only previously known retrotransposon sequences from other species as a query, the more divergent elements in grape would have been overlooked. Thus, our work expands the utility of the Pearce technique from plants with little to no available genomic sequence data available through those plants with fully characterized genomes.

Among the 24 sequences containing an identifiable portion of the 3'-end of retrotransposons, 18 were unique and six almost identical (corresponding to the *Edel *element family). Full-length retrotransposon nucleotide sequence was then identified from the grape genome by querying in BLAST with the 3'-end of the retrotransposon identified by the Pearce technique along with pre-determined total length criteria (see Materials and Methods). Clustering of the 21 different full-length retrotransposons identified by BLAST into distinct families was based on the alignment of their corresponding amino acid sequences between reverse transcriptase motifs I to VII [16]. Ten families were identified, with insertion numbers ranging from an single to 8 element insertions. The observed level of identity when comparing amino acid sequences was greater than 79% between elements clustered in the same family, while it decreased from 35–67% between elements classified within differing Ty1/*copia *families, and further drops from 12–21% between any member of the Ty1/*copia *families and *Gret1*, a unique grapevine *gypsy *element. However, within the corresponding amino acid region, the identity values we obtained are lower than those proposed by Bowen and McDonald [16] for grouping a particular element into a family (>90% identity) and into a superfamily (>75% identity). In our study, amino acid identity values alone did not suggest a clear-cut discrimination point sufficient to classify unknown elements, and a neighbor-joining phylogenetic analysis was more informative.

The ten families we identified were all related to Ty1/*copia *superfamily, as expected from a technique employing primers designed to target Ty1/*copia *RnaseH motifs. However, using the same procedure, *Tmt1*, a *Tuber *LTR-retrotransposon was isolated. Its relatedness to Ty3/*gypsy*, retrotransposon superfamily was further established [26] showing that the Pearce method is also suitable to isolate *gypsy *elements. The classification into families of the PCR-amplified fragments revealed an over-representation the two families *Gentil *and *Edel *which were further shown to have the highest copy numbers in the grape genome, whereas six families have been identified from a unique fragment among them *Noble *that showed as many copies as *Edel*. Finally seven families start with the four-nucleotide stretch TGTT. Thus, the Pearce technique may have additional biases for sequence isolation within the Ty1/*copia *group.

### Genomic fraction of the genome occupied by 13 grapevine retrotransposon families

The BLAT program was used to extract from the PN40024 genome a total of 1709 copies paralogous to the 13 canonical copies representing the families identified in this study at the most stringent criteria for seeding alignments based on sequences of 95% and greater similarity over 40 bases of length or more. BLAT also detected a small number of perfect sequence matches as short as 16 bases that we have taken in consideration. Because of the stringent identification parameters we used, the copy number of these families could be underestimated. Only copies flanked by perfect 5 bp-duplication target sites that result from repair of the integration event were considered, in order to eliminate chimerical copies that could have resulted from errors introduced during genome assemblage. No apparent conservation between 5 bp-direct repeats flanking the 10 canonical elements were observed, only predominance for A-rich sites.

Excluding *Gret1*, a total of 1,536 Ty1/*copia*-like copies belonging to the 12 families cover 1.03% of the PN40024 genome. This number can be compared to the 17,293 occurrences (5.16%) identified in the PN40024 genome by BlastX annotation or to the 56,890 occurrences (8.35%) identified by manual annotation of Ty1/*copia *superfamily [4]. The 1,536 occurrences constitute a total DNA amount of 3,771.48 Kb, which corresponds to 8.88% of 24,640.8 Kb occupied by the 17,293 occurrences and to 2.69% of 39,848.3 Kb occupied by the 56,890 occurrences. *Gret1*, the only Ty3 *gyspy *retrotransposon considered in this study covers the largest fraction of the genome, probably due to its length. The 173 identified copies covering 1,028.36 Kb (0.21%) of the PN40024 genome are a part of the 9,632 occurrences of the total fraction of Ty3/*gypsy *superfamily that cover 17,659.6 Kb and of the 14,093 occurrences covering 15,339.8 Kb identified by both annotations. Thus, the 173 occurrences correspond to 1.79% to 1.22% of the total Ty3/*gypsy *superfamily depending on the annotation method. Overall, these data indicate that these retrotransposon insertions constitute only a small fraction of the total retrotransposon DNA content of the grape genome.

Genomic distribution of copies belonging to the *ten *families is not uniform, with chromosome 16 having the highest density and chromosome 8 the lowest. However, this result has to be cautiously considered since a large number of copies belong to scaffolds that remain to be attributed to a particular chromosome. Retrotransposon density seems to be independent of the density of resistance gene analog (RGA) markers since these two chromosomes are very poor in mapped RGA loci [34]. Our results are in general agreement with the uneven distribution of transposable elements over the grape chromosomes, with insertions mainly restricted to low gene density regions [4].

### Regulation and functionality of the different retrotransposon families

A main feature of the novel families, except *Brand*, is their LTR size ranging from 147 to 373 bp, shorter that most of the plant retrotransposons previously characterized such as *Ta1, Tnt1*, and *BARE1 *with LTRs of 514, 610 and 1,829 bp, respectively. This common feature of relatively short LTRs could then characterize grapevine Ty1/*copia *retrotransposons. Seven families begin with TGTT, a four-nucleotide stretch that also starts many LTR-retrotransposons such as *Tvv1*, *Vine-1*, *Tst1, Tto1, PDR1, Ta1 *or *copia*. However, neither *Tvv1 *nor *Vine1 *sequences were captured by this application of the Pearce technique in spite of their short LTRs of 149 and 287 bp, respectively, and starting with the four-nucleotide stretch TGTT.

In the *Noble *family, copies differ by their UTL region sized from 269–695 bp. A sequence analysis determined that this size variation is essentially caused by large deletions/insertions within the internal UTL region. *Noble *family elements are similar to *Tvv1 *elements in the source of their size variation within this region [35].

One to eight copies encoding a single putatively functional *gag-pol *polyprotein were identified for three families, indicating that these families could be still autonomous and active. For the others, no autonomous copies were identified. Our *in silico *analysis ultimately showed that no full-length copies of *Vine 1*, first described truncated in its 3' ORF sequence [22] are present in the grapevine genome (Additional file 1). A subset of presumably non-autonomous retrotransposons had identical 5' and 3'-LTRs indicating that they were recently inserted at that locus. It is possible that PN40024, an accession bred to near homozygosity, has lost the corresponding active copies during the successive cycles of self-fertilization. However, these functional copies could still remain in the heterozygous parent and in other grapevine accessions as well. It is also possible that *trans *complementation can occur between autonomous and non-autonomous families, resulting in the movement of retrotransposons that do not themselves code for all of the necessary replication enzymatic machinery.

### Dating of insertion of the different families

S-SAP insertion patterns of 8 retrotransposon families on 10 *Vitis *accessions showed that these retrotransposon families are present across the *Vitis *genus. Only a few insertion sites are fixed in all accessions, which should have been maintained during speciation. Most of the scored bands are polymorphic, indicating that these families have been active after speciation across the genus. Differences in the number of bands shared in both sets can be interpreted in the light of the differences in activity level between retrotransposon families. The lower polymorphism level of *Gans *could indicate that activity of this family was confined to the distant past, whereas the high polymorphism of *Edel *could indicate more recent activity. To provide more precise insights into the evolutionary histories and activities of different families, time since insertion of full-length copies belonging to 13 families was estimated. However, the stringent parameters that we used to retrieve full-length copies has probably eliminated the most degenerated, thus the oldest, copies. In our analysis 71% of the full-length retrotransposon copies were inserted within the last 2 My, possibly thanks to a burst of retrotransposition. Finally, in a chronology of the insertions of the different families, the older *Kastel *copy being ~11 million years old is in agreement with paleo-botanical data that record the first Vitaceae fossils from the Miocene (23.0 to 5.3 My) [36].

## Conclusion

Combining a molecular genetic approach with a computer-based study, we have identified ten novel grapevine Ty1/*copia*-like retrotransposon families, none of them belonging to a retrotransposon family previously described. Our study has increased the number of grapevine retrotrotransposons from 3 to 13. Studying the characteristics of the different families, we show that at least three families could be still autonomous and active with 1–8 copies encoding a single putatively functional *gag-pol *polyprotein. We have further characterized the representation of each of the 13 grapevine retrotransposon families in the genome, including in our study *Tvv1, Vine-1 *and *Gret1*. *Vine-1 *and *Gret1 *have both been previously represented by only a single copy characterized in the genome. Altogether, we have retrieved 1,709 copies belonging to the 13 families in this study. They currently represent 1.24% of the sequenced genome, but this corresponds to only half of the amount of retrotransposon DNA present at the time of insertion. Lastly, we show the presence of 8 retrotransposon families across the *Vitis *genus, with S-SAP insertion polymorphism indicating their activity after speciation. While the amplification history of the 13 families varies both in timing and between families, a majority of copies belonging to 7 families were inserted within the last 2 My. To our knowledge, this study is the first extensive census of LTR-retrotransposons of the grapevine genome.

## Methods

### Plant material and DNA extraction

3'-end retroelement sequences were isolated from pinot noir S15. S-SAP fingerprinting was performed on a set of 10 *Vitis *accessions including 1 accession each of *Muscadinia rotundifolia*, *Vitis amurensis *and *V. riparia*, and 7 exemplars of the *V. vinifera *species, represented by PN40024, 5 accessions of cultivated grapevine varieties and 1 accession of a wild vine. Total genomic DNA extractions were performed using the Qiagen Plant extraction kit (Qiagen, Valencia, CA) according to manufacturer's protocols.

### Sequence isolation

Isolation of 3'-end retroelement sequences was performed according to Pearce et al. [25] with modifications. The first amplification was performed using both published primers: an adapter primer targeting the synthetic adapter sequence ligated to the genome fragments produced by restriction digestion paired with a biotinylated retroelement-specific primer targeting RnaseH motif 1. Following bead-based recovery of the biotinylated product, a nested amplification was performed using an adapter primer modified from the published sequence by the addition of a single selective base at the 3'-end of the primer. The modification of the adapter primer reduced the formation of products resulting from adapter to adapter amplification and increased the recovery of retroelement-containing DNA sequences. The modified adapter primer was paired with the published retroelement-specific primer targeting the downstream sequence RnaseH motif 2. PCR products were cleaned up using the QIAquick PCR purification kit (Qiagen, Valencia, CA) before being cloned using the Promega pGEM T-Easy cloning kit (Promega, Madison, WI) with Invitrogen DH5 *E. coli *cells (Invitrogen, Carlsbad, CA) according to manufacturer's protocols.

### Sequence analysis

Computer-assisted analysis of the twenty-seven 3'-end retroelement sequence fragments was performed in DNASTAR SeqManII. Full-length corresponding elements were located in the grapevine genome using Genoscope BLAST server [37] by identifying LTRs separated by 4–5 Kb along with target site duplications that mark retrotransposon insertion. They were then individually examined for position of their LTRs, UTL and ORFs in DNAsis 2.1 (Hitachi Software Engineering Co, Ltd) and Vector NTI 7 (InforMax Inc). Amino acid sequences between reverse transcriptase motifs I to VII were deduced from the nucleic acid sequences. MEGA v3.1 [38] was used to achieve multiple alignment of partial amino acid sequences by Clustal W and to draw Neighbor-Joining tree using the Poisson distance model. Amino acid sequences were compared two by two using the EMBOSS Pairwise Alignment Algorithms [39]. The 10 families were named after wines produced or vineyards cultivated in the Alsace region of France.

### Data mining

The BLAT program was used to extract the paralogous copies of the ten families from the PN40024 genome [40]. BLAT on DNA is designed to find sequences displaying stretches of 40 bases or more in length with 95% and greater similar to the query. It also finds perfect shorter sequence matches. All matches that were at least 80% of the length of the full-length insertions identified by BLAT and had a similarity level higher than 75% were considered to identify canonical copies. The nucleotide data of the 10 reported canonical copies have been deposited in Repbase database. Using *gag-pol *polyprotein sequences available from public databases, we selected amino acid sequences between reverse transcriptase motifs I to VII to build the Neighbor-Joining tree based on the Poisson distance model.

### Genomic paleontology

The insertion of full-length copies were dated based on the divergence of the 5' and 3'-LTR sequence of each copy, as proposed by San Miguel et al. [20]. LTR sequences were aligned in MEGA v3.1 and the observed divergence was corrected following the Jukes and Cantor molecular evolution model, as proposed by Vitte et al. [33]. This corrected divergence was then translated to estimate the insertion date of the elements at their present location, using the average base substitution rate derived from the grass *adh1-adh2 *region of 6.5 × 10^-9 ^substitution per site per year [41], given the assumption of a uniform mutation rate in grapevine.

### S-SAP analysis

S-SAP insertion patterns were produced largely following the protocol described by Waugh et al. [29]. LTR forward primers for 8 retrotransposon families were designed from the region of the LTR with the highest level of conservation for each family. Each LTR-specific primer was used together with the same adaptor primer Eco-AG, which had two selective nucleotides. Retrotransposon primers were IRD 800 5' labeled (MWG Biotech AG, Ebersberg, Germany) to resolve PCR fragments by electrophoresis on a 41 cm long 5.5% acrylamide gel (KB Plus 5.5% gel matrix, Li-Cor Biotechnology, Lincoln, NB) in a Li-Cor 4000 L automated DNA sequencer (Lincoln, NB). IRD41-labeled M13 fragments (50–1,206 bp) were used as size standard.

For each accession and each retrotransposon, S-SAP amplifications were performed at least twice and loaded on independent gels to establish consistency. S-SAP bands in each individual lane were detected using the RFLPscan (version 2.1) software (Scanalytics, Fairfax, VA). The distance from the retroelement-specific primer binding site within the LTR to the genomic portion of the fragment ranged from 50 bp to 106 bp. Thus, only reproducible bands greater than 125 bp in length corresponding to insertion sites were scored.

## Authors' contributions

CM participated in the design of the study, carried out the BLAT and the phylogenic analysis and helped to draft the manuscript. KG and CPM selected the system for retroelement sequence isolation, performed the molecular genetic studies and helped to draft the manuscript. FP conceived the study, carried out the BLAST and S-SAP analysis and drafted the manuscript. All authors read and approved the final manuscript.

## Supplementary Material

Additional file 1 **Global analysis of the 10 grapevine retrotransposon families** Click here for file

Additional file 2 **Sequence characteristics, location, and accession numbers of the ten canonical elements identified in the grapevine genome sequence.** Click here for file
