# Supplementary material for: Characterization of ten novel Ty1/copia-like retrotransposon families of the grapevine genome
Source: BMC Genomics. 2008 Oct 9;9:469. doi: 10.1186/1471-2164-9-469 (PMC2576258; doi:10.1186/1471-2164-9-469)
Supplement: Additional file 1 — Global analysis of the 10 grapevine retrotransposon families [file 1471-2164-9-469-S1.pdf]

Family name		*Brand*	*Cremant*	*Edel*	*Gans*	*Gentil*	*Huben*	*Kastel*	*Noble*	*Rangen*	*Wintz*	*Gret1*	*Tvv1*	*Vine-1*
**Reference copy**		KGii9-5	KGii-3-3	KGc68-1	KGii-8-2	KGii6-2	KG118-7	KGii-3-2	KGii7-2	KGii-1-3	KG116-7	AB111100	*Tvv1-VB*	AF116598
**Reference size (bp)**		5519	4807	4783	4217	4265	4941	4072	5502	5101	5278	10422	5222	2392
**Copy number**		92	110	187	94	212	91	91	185	105	126	173	103	140
**Identity (%)**	min	70.9	54.0	93.2	80.0	62.9	60.0	88.5	86.3	60.8	62.1	74.5	62.8	91.6
	med	87.75	91.05	96.6	97.1	95.7	90.7	93.7	97.3	94.4	80.35	88.3	97.4	96.9
	mean	85.74	89.69	96.35	96.08	95.35	90.93	93.60	97.25	91.78	81.68	88.95	94.57	96.59
	max	100	100	99.9	100	100	100	100	100	100	100	100	100	99
**Size (bp)**	min	22	17	74	19	16	17	16	26	16	20	21	26	20
	med	633	45	4407	253	3088	2922	3623	5122	2180	616	6290	245	292
	mean	1585	589	4001	1520	2834	2589	3180	4229	2590	903	5944	2117	1127
	max	9177	6921	18308	4960	4852	5824	7410	7040	7113	5396	10423	6963	2573
**Size (% reference)**	min	0.4	0.4	1.5	0.5	0.4	0.3	0.3	0.5	0.3	0.3	0.2	0.5	0.8
	med	11.5	0.9	92.1	6.0	72.4	59.1	88.9	93.1	42.7	11.7	60.4	4.7	12.2
	mean	28.7	12.3	83.6	36.1	66.5	52.4	78.1	76.9	50.8	17.1	57.0	40.5	47.1
	max	166.3	144.0	382.8	117.6	113.8	117.9	182.0	128.0	139.4	102.2	100.0	133.3	107.6
**Number of insertions per chromosome in PN40024**	chr 1	1	5	7	5	15	7	3	11	3	9	4	3	
	chr 2	4		4		7	3	4	9	3	11	7	1	4
	chr 3		1	4		8	3	3	8	3	1	2		
	chr 4	8	1	9		8	1	6	10	1	6	5	3	2
	chr 5	1	2	8	17	10	7	4	7		5	10	8	7
	chr 6	6	5	5	3	5	1	2	5	7	2	16	9	3
	chr 7		4	2		9		4	3		1	7	11	9
	chr 8	1	2	5		5		3	4	9	2	6	13	4
	chr 9	3		10	3	12	4	6	5	8	1	10		6
	chr 10	4	6	8	3	6	5	1	6	8	8	3	4	1
	chr 11	4	8	2		4	3	5	7	1	9	3	2	4
	chr 12	12	7	9	9	10	3	2	6	7	10	5	4	7
	chr 13	8	2	9	3	2	2		8	4	4	4	2	13
	chr 14	10	8	6	3	14	9	5	10	9	5	6	5	5
	chr 15	1	9	8	9	9		4	4		2	5		
	chr 16	3	11	7	3	5	1	5	9	3	3	1	2	6
	chr 17	4	5	7	3	3	3	4	4	2	5	15	3	5
	chr 18	5	11	10	8	16	4	8	18	4	7	13	8	10
	chr 19		6	7	2	13	1	1	3	4	9	8	2	8
	Unknown	17	17	60	23	51	34	21	48	29	26	43	23	46
**Theoritical inserted DNA (Kb)**		507.75	528.77	894.42	396.40	904.18	449.63	370.55	1017.87	535.61	665.03	1803.1	537.87	334.88
**Actual coverage (Kb)**		145.86	64.80	748.11	142.92	600.89	235.59	289.34	782.34	271.94	113.75	1028.36	218.08	157.83
**% PN40024 genome**		0.03	0.01	0.15	0.3	0.12	0.05	0.06	0.16	0.06	0.02	0.21	0.04	0.03
