# Supplementary material for: Characterization of ten novel Ty1/copia-like retrotransposon families of the grapevine genome
Source: BMC Genomics. 2008 Oct 9;9:469. doi: 10.1186/1471-2164-9-469 (PMC2576258; doi:10.1186/1471-2164-9-469)
Supplement: Additional file 2 — Sequence characteristics, location, and accession numbers of the ten canonical elements identified in the grapevine genome sequence. [file 1471-2164-9-469-S2.pdf]

Reference element	Repbase nomenclature	Element size (bp)	5'LTR - 3' LTR (bp)	Internal domain (bp)	LTR identity %	Direct flanking repeat	LTR end dinucleotides	UTL region (bp)	Gag-pol (aa)	Location		
										Chromosome	Scaffold N°	NCBI accession n°
**Brand-B01**	Copia-29_VV	5519	500-502	4517	94.4	aatat	TGTT/AACA			unknown	scaffold_163	CU459380
**Cremant-B05**	Copia-30_VV	4809	245-244	4320	99.2	atata	TAAC/GTCA	35	1346	chr11	scaffold_118	CU459335
**Edel-B05**	Copia-31_VV	4774	373-373	4028	100	ggagc	TGTT/TACA	39	1298	chr8	scaffold_23	CU459240
**Gans-B08**	Copia-32_VV	4922	247-247	4428	100	gtagt	TGTT/TACA			chr12	scaffold_78	CU459295
**Gentil-B05**	Copia-33_VV	4251	297-297	3657	99.7	gttgt	TGTT/AAGA			chr9	scaffold_33	CU459250
**Huben-B01**	Copia-1_VV*	4944	206-206	4532	100	atata	TGTG/TTCA			chr14	scaffold_21	CU459238
**Kastel-B06**	Copia-18_VV*	4086	249-231	3606	89.2	gtaaa	TGTT/GTCA			chr5	scaffold_67	CU459284
**Noble-AF04**	Copia-3_VV*	5485	200-200	5085	99.5	gaagt	TGTT/TTCA	617	1486	chr5	scaffold_58	CU459275
**Rangen-B07**	Copia-34_VV	5020	168-147	4705	91.9	aatgc	TGTT/AACA			chr19	scaffold_35	CU459252
**Wintz-B01**	Copia-35_VV	5281	291-291	4999	99.3	aaaac	TGTG/GTCA			chr2	scaffold_140	CU459357
